# Ibrutinib response in cutaneous transformed lymphoplasmacytic lymphoma

**DOI:** 10.1002/jha2.253

**Published:** 2021-06-20

**Authors:** Kaleigh E. Lindholm, Peter A. Forsberg, Mark D. Ewalt

**Affiliations:** ^1^ Department of Pathology University of Colorado Anschutz Medical Campus Aurora Colorado USA; ^2^ Department of Medicine Division of Hematology, University of Colorado Anschutz Medical Campus Aurora Colorado USA; ^3^ Department of Pathology Memorial Sloan Kettering Cancer Center New York City New York USA

Lymphoplasmacytic lymphoma (LPL) accounts for approximately 2% of hematologic malignancies [1, 2] and classically involves the bone marrow and peripheral blood, with less common involvement of the lymph nodes and spleen. LPL is often associated with a monoclonal paraprotein, usually IgM, which when symptomatic is referred to as the clinical syndrome Waldenström macroglobulinemia (WM). Disease manifestations may be due to the paraprotein or less commonly, secondary to tissue infiltration by lymphoma cells that consist of mature B‐lymphocytes, plasmacytoid lymphocytes, and plasma cells. Cutaneous involvement is uncommon in LPL, most often related to the IgM paraprotein and can range from bullous dermatoses to crystal‐storing histiocytosis to cutaneous amyloidosis [2]. Dermal infiltration by lymphoma cells is extremely rare, and when this does occur, the neoplastic cells show varying growth patterns including interstitial, nodular, and diffuse dermal infiltrates. Over 90% of cases of LPL harbor the myeloid differentiation primary response 88 gene (*MYD88*) p.L265P mutation [1, 2, 3]. LPL is generally indolent with a median survival of 5–10 years; however, the uncommon transformation to diffuse large B‐cell lymphoma (DLCBL) portends a poor prognosis with a median survival of 2 years [1, 2, 4]. Furthermore, infiltration of the skin by transformed LPL is more common than in nontransformed disease with a recent review finding cutaneous involvement in 12% of histologically transformed cases [5].

We present a 78‐year‐old male with a history of WM diagnosed in 2014 when a bone marrow biopsy showed LPL (Figure 2A,B), which harbored the *MYD88* p.L265P mutation. He underwent six cycles of bendamustine/rituximab and achieved complete remission. In 2019, he returned with a rising IgM level (859 mg/dl) in the setting of multiple new 3–4 cm subcutaneous nodules on his lower extremities (Figure 1A). A biopsy of one lesion revealed a B‐cell lymphoma with plasmacytic differentiation and aggressive features including blastoid morphology, which were positive for CD20, kappa‐restricted, positive for IgM, and showed a high proliferation rate by Ki‐67/MIB1 (Figure 2C–I). Molecular analysis revealed the MYD88 p.L265P mutation by allele‐specific quantitative real‐time PCR analysis. Given these characteristics and the patient's history of LPL with IgM kappa paraprotein, the findings were consistent with cutaneous large cell transformation of LPL. Given the patient's age and difficulty tolerating prior chemoimmunotherapy, the decision was made to trial therapy with ibrutinib monotherapy. Within 1 week of initiation of ibrutinib therapy, the subcutaneous lesions had decreased in size (Figure 1B). At 1 month following initiation of therapy, the patient's serum IgM level had decreased and the skin lesions showed further marked improvement with complete flattening and lightening of the lesions (Figure 1C). The lesions remained flat for 7.5 months following therapy initiation (Figure 1D). At most recent follow‐up, the patient has sustained disease control at 18 months from initiation of ibrutinib therapy.

**FIGURE 1:**
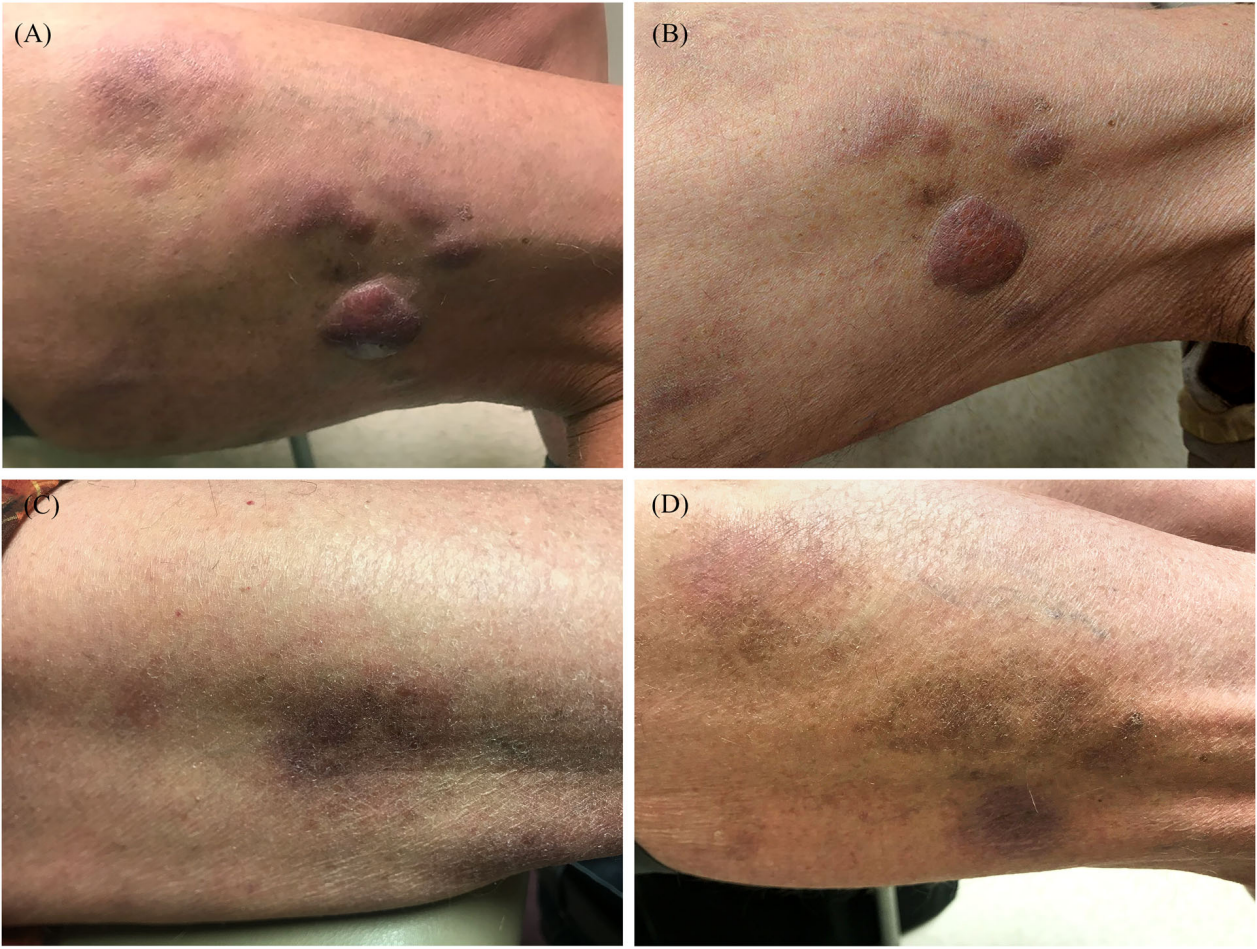
Clinical appearance of patient's skin lesion on the right lateral thigh (A) at diagnosis, (B) following 1 week of ibrutinib therapy, (C) following 1 month of ibrutinib therapy, and (D) following 7.5 months of ibrutinib therapy

**FIGURE 2:**
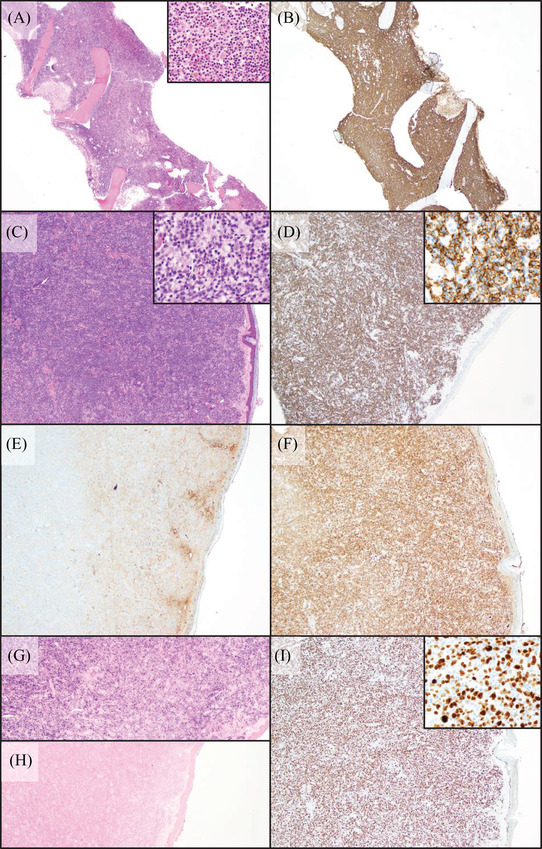
Histologic sections of bone marrow (A) H&E, (B) CD20 and skin, (C) H&E, (D) CD20 IHC, (E) IgG IHC, (F) IgM IHC, (G) kappa ISH, (H) lambda ISH, and (I) Ki‐67 IHC. All images shown are at 4× magnification, with insets at 60× magnification where provided. H&E, hematoxylin and eosin; IHC, immunohistochemistry; ISH, in situ hybridization

In nearly all LPL cases, the *MYD88* p.L265P mutation is present. Wildtype MYD88 is activated by toll‐like receptors and interleukin‐1 pathways and subsequently activates downstream NF‐kB signaling [6, 7]. The p.L265P mutation causes the mutant MYD88 to form a constitutively active complex with BTK, with subsequent downstream hyperactivation of the NF‐kB pathway and lymphomagenesis [6, 7]. In recent years, small‐molecule inhibitors have become increasingly common in the treatment of hematopoietic neoplasms. Ibrutinib, a BTK inhibitor, is one such compound that has been approved for use in several B‐cell neoplasms, including WM/LPL for which it was approved in 2015. Ibrutinib functions by forming an irreversible covalent bond with a cysteine residue within the ATP binding domain of BTK, decreasing downstream NF‐kB activation, increasing apoptosis, and decreasing cell proliferation [8, 9, 10].

While ibrutinib is a common therapy for LPL and also shows some efficacy in certain subtypes of DLBCL, we know of only two papers addressing the use of ibrutinib in transformed LPL. The first is of a 63‐year‐old female originally presenting with LPL within the bone marrow who later developed CNS disease and a submandibular lymph node, the latter of which demonstrated DLBCL by histology [11]. Interestingly, molecular testing showed the presence of *MYD88* p.L265P mutation in the original bone marrow specimen; however, it was lacking in the lymph node [11]. Clonality analysis testing revealed the bone marrow and the lymph node to be the same B‐cell clone. The authors suggested there was loss of the mutation with high‐grade transformation of the LPL [11]. Nonetheless, ibrutinib therapy was initiated. Unfortunately, while the patient showed a promising initial response, she relapsed 5 months later and subsequently passed away [11]. Given the absence of the *MYD88* mutation in the transformed sample, it is difficult to ascribe this initial response to effectiveness of that therapy in all stages of LPL as opposed to representing a case of the known phenomena of ibrutinib response in non‐GC DLBCL and the possibility that the LPL and DLBCL in that sample arose from the same ancestral clone through activation of different pathways [11]. The second paper consists of a study assessing ibrutinib monotherapy in patients with relapsed or refractory DLBCL. Of the 20 patients included, two were originally diagnosed with LPL [12]. The overall response rate was 35% with an overall survival of 22.4 months. Interestingly, the response was not found to depend on the original lymphoma subtype [12]. However, one patient discontinued therapy early and one patient passed away while on therapy; the specific details on the two LPL patients including the presence or absence of the MYD88 L265P mutation were not given.

In conclusion, we report the first known case of a patient with molecularly confirmed high‐grade transformation of a previously diagnosed lymphoplasmacytic lymphoma who showed marked rapid and sustained control of his disease with initiation of targeted therapy. Historically, high‐intensity chemotherapy has been the most common therapy utilized following transformation. Small‐molecule inhibitors such as ibrutinib, though widely used in the setting of WM/LPL, have not been extensively studied in the setting of transformation. With their generally well‐tolerated side effect profile compared to high‐intensity chemotherapy, small‐molecule BTK inhibitors may be a reasonable treatment consideration in certain patients showing high‐grade transformation who are not considered to be ideal candidates for more intensive therapy due to age, frailty, or the presence of significant comorbidities or who have failed standard approaches.

## CONFLICT OF INTEREST

The authors declare that there is no conflict of interest.

## Data Availability

Data sharing not applicable to this article as no datasets were generated or analyzed during the current study.
